# Occupational Dermatitis*Extracted from “Around the Table” in *Dental Items of Interest*, January, 1922.

**Published:** 1922-02

**Authors:** A. S. Walker

**Affiliations:** New York


### OCCUPATIONAL DERMATITIS*

BY A. S. WALKER, M.D., NEW YORK

"The attack of metol poisoning from which I suffered lasted all through the winter of 1919-1920 and until August, 1921. During my vacation I dispensed with water for washing the hands as much as possible, substituting oil for that purpose, and upon my return to work I used cold cream for routine hand cleansing, resorting to the use of rubber gloves for pulp operations. Rubber gloves should be used as little as possible by one suffering

* Extracted from "Around the Table" in *Dental Items of Interest,* January, 1922.

from this form of dermatitis on account of the perspiring induced by wearing them. My condition was entirely cured before I corresponded with the Eastman people, so, of course, none of the remedies suggested by them were used by me.''

''During the past eighteen months a form of eczema has made its appearance on the thumbs and fingers of dentists, and their assistants who have been developing the X-ray films. The first symptom is an itching of the affected parts, sometimes so severe as to prevent sleep. This is followed in a few days by breaks and peeling of the epidermis. In severe cases this may be followed by bleeding, and failure to heal for several months, the slow healing being due to frequent washing of the hands. So far as I know none of our dermatologists in New York have traced the cause of the trouble. One specialist has called it 'chemical eczema.' Having been a victim myself, I have been more than ordinarily interested, and am satisfied that it is caused by some ingredient of the developer. Would it be the 'hydrochinon'? Every case that has come to my notice was on the hands of those who have been developing. Where it has made its appearance on one hand only, it was the hand immersed in the developing solution. Some persons seem to have a complete immunity. My nurse is an example. It has been suggested that immersing the hands in water before putting them in the developer would prevent trouble. I am writing this to you to ascertain if this trouble has come to your notice. Will you be kind enough to let me have your views?''

The Eastman Kodak Company replied in the following exceedingly comprehensive manner, and this communication becomes an important contribution to the study of this subject:

''We are very sorry to learn from your letter of January 27 that you have been troubled with what is commonly known as 'metol poisoning.'

"From a digest of a large mass of information on the subject, it would appear that the chemical poisoning is secondary to the primary action of the alkali in the developer. This partially dissolves the skin and causes cracks to form, thus exposing the under layers of the epidermis to attack by the reducing of the developer and the oxidation products of the same.

"The following facts have been fairly well established: That potassium carbonate in a developer is less liable to cause the trouble than sodium carbonate. This is probably owing to the deliquescent nature of the former. Sodium carbonate effloresces on the hands and causes cracking of the skin.

"That a non-alkaline developer like amidol does not give much trouble, though a few cases of poisoning with this developer are on record. In this case also, sodium sulphate efflorescing on the hands opens up the pores in the same way as sodium carbonate.

"That bathing the hands in weak acid at intervals during development is an almost certain preventive, and several cases are recorded where a cure has been effected by rubbing well several times a day with the following:

Acetic Acid No. 8—1 ounce.
Water—2 ounces.
Sodium Chloride—1 tablespoon.
Two per cent. H Cl is also recommended.

"If an acid stop bath is used between development and fixation, the above bathing of the hands is done unconsciously. Several persons have been cured in this way.

"The effective use of acid as a preventive is, no doubt, due to its neutralizing action on the alkali left in the skin, and which is very difficult to remove by washing, especially when the skin is at all cracked.

"That bathing the hands in potassium permanganate and subsequently removing the stain with oxalic acid has been of benefit in many cases. The action here is both to neutralize the alkali and remove any oxidation products of the developer which may have penetrated the cracked skin.

"The following solutions are suggested:

| | | |
|---|---|---|
| A— | Potassium permanganate..... | 2 parts |
| | Concentrated sulphuric...... | 2 parts |
| | Water ..................... | 1,000 parts |
| B— | Oxalic acid................. | 20 parts |
| | Water ..................... | 1,000 parts |

"Of the numerous oily preparations which have been recommended, either 'Hazeline Cream' of Messrs. Burrough Wellcome, 'Cold Cream,' 'Zinc Ointment,' or the following:

| | |
|---|---|
| Ichthyol .......................... | 1 oz. |
| Resorcin .......................... | 1 oz. |
| Glycerine .......................... | 1 oz. |
| Zinc oxide .......................... | ½ oz. |
| White paraffin ointment............. | 6 oz. |

have been found to give relief and in many cases effect a cure. The action of the oily preparation is to prevent access of the developer to the under layers of the skin."

I give now my personal experience because it may shed more light upon a condition that is none too well comprehended. My first attack was upon the tips of the index and second fingers of the right hand. I mention these localities because of a coincidence which at first led my mind along the wrong track, and this but shows the need of sound logic when studying etiologic factors. In my own mind I attributed the disturbance to the X-ray because the only places attacked were exactly the only parts of my body exposed to the X-ray. At that time I was using a very small aperture with my radiographic machine, and I was using these two fingers for holding films in the mouth.

I was sufficiently disturbed to travel down to Philadelphia to seek the advice of a well-known dermatologist, who had had considerable experience with X-ray burns in the early days. But this gentleman promptly declared it was not the X-ray. Then he asked, "Do you use formalin?" Receiving a negative reply, he asked, "Do you use metol?" When I admitted this he at once stated that it was a case of metol poisoning.

This specialist gave me a salve, which, as I recall, contained salicylic acid, which was probably intended to cut down the growths which extruded from beneath the nails of both fingers. This was applied at night and covered with kid-glove fingers. I also used rubber finger cots when developing.

The point of interest here is that it was exactly the tips of these two fingers that I had been immersing in my developers, when developing films, as at that time I was using the old-fashioned tray method.

The use of the salve brought some improvement, and the trouble disappeared during the summer vacation, proving the old adage that "almost any danger can be avoided by presence of mind and—absence of body." The vacation brought abstinence from the use of the developer, and probably did more to effect a cure than the salve.

During the next winter I abandoned metol and used hydrochinon, because I recalled that in my more than fifteen years' experience in developing large plates in pictorial photography I had used hydrochinon, freely immersing my hands in it at all times without disturbance of any kind. However, I chanced upon a formula which develops very rapidly, and produces sharp contrasts. It is a very concentrated solution, strong in powerful caustic alkalis. I resorted to a deep holder for the developing solution and handled the films with clips. In spite of these precautions, I presume that the developer frequently reached my fingers. It is noteworthy that whereas in the former method of developing it was the ends of the fingers and the palmer surface of the tips that were affected, with the new method it was the inner, or I may say the under surfaces, of the index and second fingers that suffered. By under, I mean the side that was downward when holding and agitating the films in the solutions.

With this highly concentrated and rapid developer, it was essential to thoroughly wash the films, before immersing them in the fixing bath. To do this I would pass the film to the left hand and wash it under the full flow of water from the faucet. I supposed that this procedure safeguarded that hand at least; but this did not prove to be the case, and the Eastman Company have now provided us with a better method, the use of the acid stop bath.

During that winter I suffered slightly on the left hand, but very intensely on the right, chiefly the two first fingers. The description given by Dr. Lane of procain dermatitis, and that given by Dr. Walker, both aptly describe my experiences. A few clinical facts, however, seem worthy of mention.

The little vesicles form and grow higher till they finally break. At this time a small magnifying glass will disclose minute areas entirely denuded of skin. These are highly sensitive and painful. The epidermis dies and rolls up, at which time it feels like so many knives cutting into the unprotected tissue below. Later on, fissures form, and as these fissures become extremely painful let me here mention the treatment which has proven most satisfactory to me. As soon as a fissure is noted, close it with adhesive plaster. This gives almost instant relief from pain, and hastens healing, whereas if unprotected the fissure grows longer and deeper.

Dr. Walker advises against washing, but my own feeling is that the Eastman Company have given the best explanation of the trouble in attributing it to the solvent action of the alkalis upon the epidermis. Hence, I would advise caution in selecting a soap, and would recommend Armour's super-fatted soap.

During that period when the itching is most excruciating, there is a great temptation in drying the hands to use a rough towel vigorously. This is a form of scratching, and while it produces a sort of temporary satisfaction, it is temporary indeed, because it hastens the breaking down of the vesicles, and causes the minute denudation. The itching is exchanged for a painful burning. Hence, a soft towel is best.

The use of rubber gloves, as Dr. Walker states, is unsatisfactory for long operations such as root canal work, but can be recommended for developing. But the technic must be perfect and rigidly adhered to. I returned from the country this autumn with my fingers perfectly cured. I resorted to finger cots, and all went well until by chance I used my finger cots reversed, so that the side which had previously been exposed to the developing solution, was now against the skin. Within three days the dermatitis appeared, and has not been entirely cured even now, three months later.

The point in regard to rubber gloves is that there must be no break in the glove which would permit solutions to enter. This means a thorough powdering of the inner side. For this purpose talcum is commonly used, but oleate of zinc is said to be even better. There is a glove now on the market with a roughened exterior and a smooth inner surface. This is the most satisfactory in use, and it becomes easy after washing or boiling a glove to make sure that the outside of the glove is not used as the inside.

This winter my fingers became so bad that I finally consulted Dr. Fred Wise of this city, who gave me the following salve:

Carboneol. ............................ʒss

Ung. diachylon (Hebra lead plaster) ad..℥j

This was almost magical in relieving the pain and curing and covering the denuded spots. But it is black and stains the fingers very badly. After two weeks, Dr. Wise substituted the black salve with the following, which is white, and does not stain:

Anthrosol ..........................ʒj

Acid salicylii .......................gr xv

Ungt. zinc oxid. ad....................℥j

This white salve is good, but not so effective as the black. Despite the staining, therefore, I recommend the former, at least "until the worst is over." In use, the salves must be massaged well and thoroughly into the affected parts, after which cotton gloves may be worn during the night.

Before closing I must again refer to the acid stop bath recommended by the Eastman Company. My own experience proves that even thorough washing under the hydrant did not suffice. But the immersion of the exposed fingers in an acid bath after the development of each film will neutralize the alkali. This, of course, is purely a prophylactic measure, and is intended to safeguard those who have never suffered from this type of dermatitis.

The surest way, then, to avoid this occupational dermatitis, is to have your office assistant do the developing. But if you do this have her use the acid stop bath.

